# Supplementary material for: Displacive-type ferroelectricity from magnetic correlations within spin-chain
Source: Sci Rep. 2014 Jul 9;4:5636. doi: 10.1038/srep05636 (PMC4088096; doi:10.1038/srep05636)
Supplement: Supplementary Information — Rietveld fitting of XRD data [file srep05636-s1.pdf]

Supplementary information

Displacive-type ferroelectricity from magnetic correlations within spin-chain

Tathamay Basu,[1] V. V. Ravi Kishore,[2] Smita Gohil,[1] Kiran Singh,[1,3] N. Mohapotra,[4] S. Bhattacharjee,[1] Babu Gonde[1], N.P. Lalla,[3] Priya Mahadevan,[2] Shankar Ghosh,[1] and E.V. Sampathkumaran[1]

[1]Tata Institute of Fundamental Research, Homi Bhabha Road, Colaba, Mumbai-400005, India

[2]S. N. Bose National Centre for Basic Sciences, Sector-III, Block-JD, Salt Lake, Kolkata - 700 098, India

[3]UGC-DAE Consortium for Scientific Research, University Campus, Khandwa Road, Indore - 452001, India

[4]School of Basic Sciences, Indian Institute of Technology Bhubaneshwar, Bhubaneshwar-751013, India

Correspondence and requests for materials should be addressed to E.V.S (sampath@mailhost.tifr.res.in)

Here, we present x-ray diffraction pattern at 30 K and show the Rietveld fitting as well for three space groups discussed in the article. The refined parameters are also in the listed in the table.

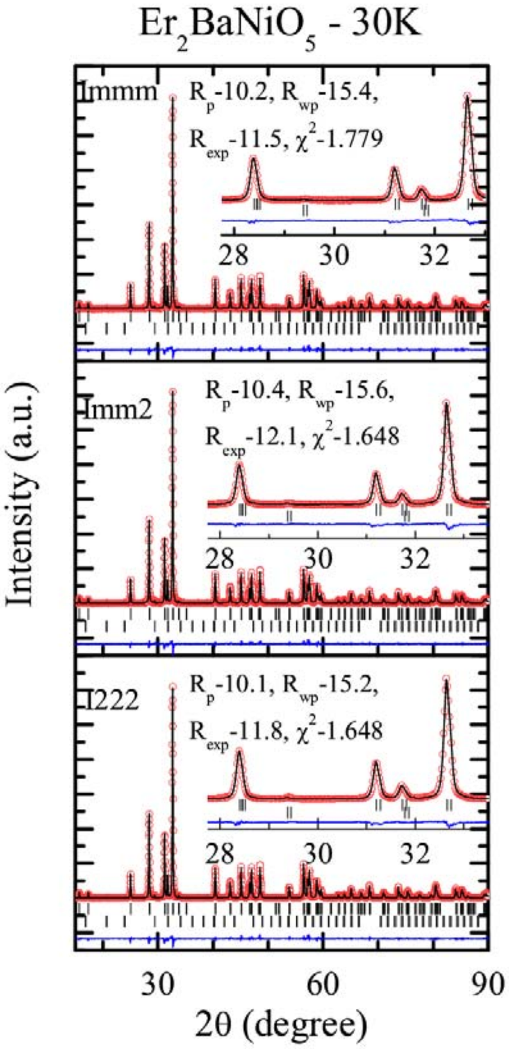

Supplementary Figure S1: The Rieveld fitted powder XRD pattern at 30 K. The fitting was carried out using FullProf program for three space groups. The parameters mentioned in the figure carry usual meaning. Observed (open red circle), calculated (continuous black line) and difference (continuous blue line) Upper vertical lines mark the Bragg positions of Er_2BaNiO_5; lower vertical lines represent Bragg positions of Er_2O_3 phase, assuming that it is present in small traces (<<2%). We also provide refined paramers below:

Supplementary Table S1: Rietveld refined structural parameters and statistical parameters

Space group: *Immm*

a= 3.7468(1) Å, b= 5.7277(1) Å, c= 11.26806(7) Å
R_p = 10.4,R_{wp} = 15.7 ,R_e = 11.9 and χ^2= 1.759

	x	y	z	B (Å^2)
Ba(2c)	0.5	0.5	0	0.24(2)
Er(4j)	0.5	0	0.202(1)	0.15(2)
Ni(2a)	0	0	0	0.29(4)
O(8l)	0	0.240(2)	0.148(3)	0.1(3)
O(2b)	0.5	0	0	0.1(4)

Space group: *Imm2*

a= 3.7466(1) Å , b= 5.7279(1) Å, c= 11.26816(7) Å
R_p = 10.2, R_{wp} = 15.2, R_e = 11.8 and χ^2= 1.664

	x	y	z	B (Å^2)
Ba(2a)	0.5	0.5	0	0.25(2)
Er1(2b)	0.5	0	0.202(1)	0.16(4)
Er2(2b)	0.5	0	0.798(3)	0.15(2)
Ni(2a)	0	0	0.002(2)	0.24(8)
O11(4d)	0	0.238(2)	0.148(3)	0.1(3)
O12(4d)	0	0.761(2)	0.852(4)	0.1(2)
O2(2b)	0.5	0	0.003(1)	0.1(1)

Space group: I222

---

a= 3.7468(4) Å,   b=5.7278(4) Å, c=11.26811(7) Å
R_p =10.1, R_{wp} =15.2, R_e =11.8 and χ^2= 1.648

	x	y	z	B (Å^2)
Ba(2c)	0.5	0.5	0	0.21(2)
Er(4j)	0.5	0	0.202(1)	0.16(2)
Ni(2a)	0	0	0	0.31(4)
O(8k)	0.000(2)	0.240(2)	0.148(3)	0.1(7)
O(2b)	0.5	0	0	0.1(7)
